# Optimal Systemic Treatment of Advanced Bladder Cancer Calls for a Multidisciplinary Approach

**DOI:** 10.3390/cancers15030559

**Published:** 2023-01-17

**Authors:** Helle Pappot, Anders Ullén

**Affiliations:** 1 Department of Oncology, Rigshospitalet, University of Copenhagen, 2100 Copenhagen, Denmark; 2 Department of Clinical Medicine, University of Copenhagen, 2200 Copenhagen, Denmark; 3 Department of Oncology-Pathology, Karolinska Institute, 17164 Stockholm, Sweden; 4 Genitourinary Oncology and Urology Unit, Department of Pelvic Cancer, Karolinska University Hospital, 14186 Stockholm, Sweden

Although the bladder cancer treatment field is expanding with several new treatments introduced in the last decade, many patients with an advanced form of the disease can expect a poor prognosis when diagnosed. Recently, the sequence of applying platinum-based chemotherapy (CHT) and immunotherapy with check-point inhibitors (ICIs) was changed from treatment with ICIs at progression or relapse after initial CHT to the use of ICIs as the maintenance therapy. This shift was based on data from a randomized clinical trial investigating avelumab versus best supportive care (BSC) in a maintenance setting for patients without progression after first-line CHT [1]. An overall survival benefit was observed in favor for maintenance avelumab, amounting to 23.8 months, versus 15.0 months for BSC [1,2]. The group of patients who progress during or after maintenance avelumab represent a rather unexplored area, calling for prospective and real-world studies. Some such patients are usually most likely considered to receive the first-in-class antibody–drug conjugate for advanced urothelial cancer, i.e., enfortumab vedotin (EV), which was recently approved by the FDA and EMA [3]. Even though EV represents an important novel treatment option for third-line patients, the response-rate is limited to 41%, as well as having a survival benefit of 4 months in comparison to chemotherapy with taxanes or vinflunine [3]. Further, no predictive clinical biomarkers for the selection of patients for this anti-Nectin-4 antibody have yet been identified, and emerging data have indicated that some patients in fact respond well to rechallenging treatment with chemotherapy after ICIs, as highlighted by Riedel et al. in the present Special Issue [4]. Given the complexity in defining the most optimal care for patients with advanced bladder cancer, we propose to recognize the treatment trajectory as a multifactorial process that relies on input from many different medical disciplines to define the most optimal and personalized management for each patient.

This Special Issue presents a multifactorial approach for advanced bladder cancer and covers both molecular issues, clinical real-world data, and the management of specific metastatic challenges. Furthermore, the present Special Issue discusses the importance of multidisciplinary approaches for optimal care.

Tumor heterogeneity is probably one of the most troublesome issues when aiming to develop modern treatment approaches in bladder cancer. Results from initial ICI studies linking PD1/PDL1 expression to clinical response have been difficult to interpret, not always showing consistency between the expression of the target protein and response [5,6,7,8,9]. When reviewing the literature, Lavallee et al. summarizes how it is important not only to better understand bladder cancer cell heterogeneity and plasticity, but also to consider tumor heterogeneity in a dynamic and adaptive fashion. The authors suggest that a dynamic approach might have the potential to improve treatment outcome and possibly overcome cancer drug tolerance [10].

Multiple biological processes might be important for the metastatic potential of bladder cancer and it is often discussed whether adjuvant therapy should be considered after cystectomy. Recent data have strengthened the introduction of ICIs, with the use of nivolumab as one routine approach for adjuvant therapy in the patient population with muscle-invasive bladder cancer at high risk for recurrence [11]. Prognostic markers superior to PD-L1 and that may help us understanding the differentiation of bladder cancer trajectories better are, however, warranted; thus, patients can be advised on a biological basis when deciding to take on long treatment series, which can hamper their health-related quality of life. Although the plasminogen activation system cannot be targeted with specific therapies, it is noteworthy how components of this enzyme system, known to be involved in both invasion and metastasis, can predict relapse and survival in bladder cancer [12]. Before implementation into routine care, prospective validated studies are needed.

Since no established biomarkers exist for the prediction of response, Sjödahl et al. investigated whether, at present, there is knowledge on molecular subtypes that would allow for selecting patients with muscle-invasive bladder cancer for neoadjuvant chemotherapy (NAC) [13]. In a narrative review in this Special Issue, Sjödahl and colleagues argue that several explorative studies have indicated that molecular subtyping may be predictive of a NAC response, but that studies of better quality testing prespecified hypotheses in prospective designs are needed before this approach is taken into clinical practice [14].

All clinicians working with advanced bladder cancer strive at achieving the best outcome for patients. When this cannot be reached by using biological parameters, such as biomarkers or molecular subtypes, clinical parameters, such as the distribution of metastatic patterns, may be considered to individualize treatment and delay the onset of systemic treatment. Reviewing the literature, Longo et al. showed how metastasis-directed radiation therapy can be a potential treatment option for selected patients with oligometastatic disease. Results should be interpreted with caution however, since these data, albeit in the form of a review, are based on a limited number of patients, *n* = 158 [15].

With the aim of improving survival and limited treatment possibilities, an idea surfaced, where drugs initially registered for a different use are tested in treatment sequences in a novel indication. Riedel et al. analyzed the effect of the vinca alkaloid vinflunine in for third-line treatment following previous treatment with CHT and a mix of various PD1/PDL1 ICIs. Vinflunine was originally approved as a second-line postplatinum treatment before the introduction of ICI [4]. In a German multicenter cohort, it was demonstrated how at times, unexpected clinical activity could be achieved in third- or later-line treatments post-ICI. The biological basis for these observations is unclear however, and, most importantly, whether the efficacy varies among different specific PD1 and PD-L1 compounds.

Altogether, the field of bladder cancer treatment and management is rapidly emerging with novel knowledge on predictive and prognostic markers, molecular subtyping, the differentiated use of NAC, the possibility of applying known drugs in novel settings, and the putative treatment of oligometastatic disease with stereotactic radiotherapy approaches. Finding the most optimal method for making use of the palette of these principally different approaches a multidisciplinary approach in clinical practice is called for. This is also what the Spanish Oncology Genitourinary Multidisciplinary Working Group [16] has suggested. Based on a thorough description of issues to be discussed and specialists to be involved regarding the treatment of bladder cancer patients, it is suggested that a multidisciplinary approach using multidisciplinary conferences could become a cornerstone in the future optimal care and treatment of every bladder cancer individual.

This present Special Issue offers significant viewpoints on several important topics in the field of the systemic treatment of advanced bladder cancer, with articles published likely to stimulate future valuable improvements in the cancer therapy of this disease.
